# Intuition and exponential growth: bias and the roles of parameterization and complexity

**DOI:** 10.1007/s00591-021-00306-7

**Published:** 2021-08-25

**Authors:** Martin Schonger, Daniela Sele

**Affiliations:** 1 Lucerne University of Applied Sciences and Arts, Lucerne, Switzerland; 2 Swiss Federal Institute of Technology Zurich, Zurich, Switzerland

**Keywords:** Exponential Growth Bias, Intuition, Framing, Heuristics, Numeracy, Didactics of mathematics

## Abstract

Exponential growth bias is the phenomenon that humans intuitively underestimate exponential growth. This article reports on an experiment where treatments differ in the parameterization of growth: Exponential growth is communicated to one group in terms of growth rates, and in terms of doubling times to the other. Exponential growth bias is much smaller when doubling times are employed. Considering that in many applications, individuals face a choice between different growth rates, rather than between exponential growth and zero growth, we ask a question where growth is reduced from high to low. Subjects vastly underestimate the effect of this reduction, though less so in the parameterization using doubling times. The answers to this question are more severely biased than one would expect from the answers to the exponential growth questions. These biases emerge despite the sample being highly educated and exhibiting awareness of exponential growth bias. Implications for teaching, the usefulness of heuristics, and policy are discussed.

## Introduction

Exponential growth is an astounding and fascinating phenomenon even, or perhaps especially, for the numerate. Many natural, technical, social and economic processes can be described by formal models using exponential functions—the uptake of fashion trends or the adoption of new technologies, the growth of bacteria populations or the spread of contagious diseases, the growth of an asset with compound interest, population growth, nuclear chain reactions or environmental processes like the stock of pollutants such as chlorofluorocarbons or carbon dioxide. Such phenomena can be captured by models of exponential growth, at least for some periods of time.^1^ Yet, despite the prevalence of exponential processes in our world, humans tend to underestimate exponential growth, a phenomenon referred to as *exponential growth bias*.

Perhaps the oldest and most famous illustration of both how astounding exponential growth is, and of the human tendency to underestimate it, is the legend about the grains of rice on the chessboard [6, 13, 17]. In this tale, a wealthy ruler wants to reward the inventor of chess for his invention of the famous game by granting him a wish. The inventor cleverly and seemingly humbly asks for a few grains of rice, the amount to be calculated as follows: a single grain of rice should be placed on the first square of the chessboard, two grains on the second square, four on the third, and so on, doubling the amount with every square, until the final and sixty-fourth square is reached. The ruler, believing this to be an exceedingly modest request, accepts without hesitation—only to realize that the entire harvest in his dominion would not be enough to fulfil it: The number of grains needed to fill all 64 squares is $2^{64}-1$. This amount vastly exceeds even the contemporary global harvest. However, we need not rely on legend or introspection to find that exponential growth bias is widespread. In recent decades, experimental and empirical research has documented the phenomenon in a variety of domains, including plant growth [25], pollution [23, 24] economic growth [4], and financial decision-making [1, 4, 7, 8, 12, 15, 16, 18]. During the Covid-19 pandemic, exponential growth bias has received both renewed scientific [14, 19] and media [2, 5, 26] attention.

While it is well-documented that humans underestimate exponential growth, little is known about whether this underestimation possesses certain regularities. Some believe that humans think linearly, while others propose that they linearize, and yet others believe that such descriptions are too restrictive. Previous research has tried to capture people’s beliefs by using formal *as-if* models. *As-if* models stand in contrast to *as-is* models which claim to describe a person’s thought process when thinking about a problem. By contrast, *as-if* models merely try to predict beliefs or behavior. Consider the following given exponential function $f\colon R^{+}\rightarrow R^{+}$ with $f\left(t\right)=\kappa \left(1+\rho \right)^{t}$, where $\kappa > 0$ is the initial value and $\rho > 0$ the growth rate. An individual’s beliefs about *f* are then described by a belief function $b\colon R^{+}\rightarrow R^{+}$. If $b\left(t\right)=f(t)$ for all *t*, then the individual’s beliefs are completely accurate. Wagenaar and Sagaria [23] propose $b\left(t\right)=\alpha \kappa \left(1+\rho \right)^{\beta t}$, where $\alpha \geq 1$, and $0< \beta \leq 1$. An unbiased person has $\alpha =\beta =1$. The special case where $\alpha =1$ is examined by Stango and Zinman [20]. Levy and Tasoff [15] propose that beliefs can be captured by a convex combination of linear and exponential growth: $b\left(t\right)=\alpha \kappa \left(1+\rho \right)^{t}+\left(1-\alpha \right)\kappa \left(1+t\rho \right)$, where $0\leq \alpha \leq 1$. At the extremes, a person with $\alpha =0$ believes that growth is linear, while for $\alpha =1$ her beliefs are accurate.

To learn more about human biases regarding exponential growth we are conducting a research program that studies a variety of contexts, including financial decision-making, intertemporal decision-making regarding innovation and infectious disease spread. Study settings include mail surveys, laboratory and online experiments, and students or representative household samples as subjects. In this article, we report on an experiment that was conducted during the first wave of the Covid-19 pandemic in Switzerland. The data from this experiment were first analysed in Schonger and Sele [19], this paper replicates parts of that analysis and offers additional analyses and insights. The paper adds a formal model and test of the hypothesis that in questions about changing growth rates exponential growth bias is neither attenuated nor increased, a discussion of popular misconceptions of exponential growth, and tentative suggestions for teaching and policy-making.

In the following, we focus on three main questions. First, whether and to what extent highly educated subjects exhibit exponential growth bias in a context where exponential growth, and the potential for bias, was salient. Second, whether and how exponential growth bias is affected by *framing*. Framing refers to different ways of communicating identical information, with different parameterizations being one example. Third, whether and how beliefs on exponential growth are affected when exponential questions are embedded in a complex context.

Relating to the first question, the study took place during the first wave of the Covid-19 pandemic, when exponential growth and the dangers of its underestimation were made salient by the media. Subjects were recruited from Swiss universities. Students from STEM fields such as mathematics or physics were excluded. Law, medicine and architecture were the most frequent fields. Subjects can hence be thought of as the kind of people who might one day hold elected office, sit on a judicial bench, staff our hospitals, or design our buildings.

The second research question is motivated by the observation that different ways of asking a hard question often prove helpful in finding a solution. Moreover, psychologists and behavioral economists have found that people respond differently to a different framing of one and the same question [3, 9–11, 21, 22]. For exponential growth, a naturally occurring framing is the choice of parameterization in growth rates vs. doubling times.

The third research question starts from the observation that in most applications, the problem does not merely consist of calculating a future value of an exponential function. As an example of a more complex problem, we ask subjects about the impact of a decrease in the growth rate.

## Study design

Subjects are given a hypothetical scenario in which a country faces an exponentially growing infectious disease. Initially, the country has 974 cases of the infectious disease. Subjects are randomly divided into two groups, which are given the information about this exponential growth process in two different frames: The first frame, given to Group R, communicates exponential growth in terms of the growth rate. The second frame, given to Group D, communicates exponential growth in terms of the doubling time. The unit of time in both cases is days, as commonly used in this context. The exponential functions used in the different frames are identical except for negligible rounding errors. In the study, subjects are asked for their intuitive understanding and thus to refrain from the use of calculators or other aids. There is a non-contingent participation fee.^2^

The experiment uses three main questions, two of which describe the exponential spread of the infectious disease (the high and low exponential growth questions), and one which describes the impact of mitigation measures taken to slow the disease spread. All three questions ask subjects to consider how the situation in the country will be in 30 days. In the low exponential growth question, subjects in Group R are asked for the number of cases after 30 days at the (relatively) low growth rate of 9% per day. Re-parameterizing this information, subjects in Group D are given a doubling time of 8 days. Due to rounding error, there is a slight difference in the speed of growth, resulting in a, for present purposes, negligible difference in the true values after 30 days; 12,923 cases in the parameterization using growth rates and 13,105 cases when using doubling times. Table 1 gives the low exponential growth question for both parameterizations.

**Table 1:** Low exponential growth question

*1a. Parameterization in terms of the daily growth rate (Group R)*
“In a country, 974 people have been infected so far. The number of infected people grows by 9% per day. How many people will be infected in 30 days?”
*1b. Parameterization in terms of doubling time in days (Group D)*
“In a country, 974 people have been infected so far. The number of infected people doubles every 8 days. How many people will be infected in 30 days?”

In the high exponential growth question, subjects are asked for the number of cases after 30 days at the high growth rate of 26% per day resp. at a doubling time of 3 days. The true values after 30 days are about a million cases in both parameterizations, 999,253 when using growth rates, and 997,376 cases when using doubling times. Table 2 gives the high exponential growth question for both parameterizations.

**Table 2:** High exponential growth question

*2a. Parameterization in terms of the daily growth rate (Group R)*
“In a country, 974 people have been infected so far. The number of infected people grows by 26% per day. How many people will be infected in 30 days?”
*2b. Parameterization in terms of doubling time in days (Group D)*
“In a country, 974 people have been infected so far. The number of infected people doubles every 3 days. How many people will be infected in 30 days?”

The third question considers the scenario where exponential growth can be reduced from a high to a low growth rate. It is constructed from the high and low exponential growth questions. It asks subjects directly how many cases can be avoided if the country implements a policy that lowers the exponential growth rate from the high growth rate to the low growth rate. We refer to this question as the *mitigation question*, Table 3 gives it in both parameterizations. In the parameterization using growth rates, the correct answer is 986,330 cases that can be avoided, and in the parameterization using doubling times 984,271.

**Table 3:** Mitigation question (reducing exponential growth)

*3a. Parameterization in terms of the daily growth rate (Group R)*
“In a country, 974 people have been infected so far. The number of infected people grows by 26% daily. The country aims to have as few infected people as possible in 30 days. Therefore, the adoption of measures such as increased hand-washing and social distancing is being discussed. With these measures, the number of infected people would grow at only 9% per day. How many infections could be avoided in the following 30 days with these measures?”
*3b. Parameterization in terms of doubling time in days (Group D)*
“In a country, 974 people have been infected so far. The number of infected people doubles every 3 days. The country aims to have as few infected people as possible in 30 days. Therefore, the adoption of measures such as increased hand-washing and social distancing is being discussed. With these measures, the number of infected people would double only every 8 days. How many infections could be avoided in the following 30 days with these measures?”

Subjects are not told in advance that there are three questions, nor that the mitigation question is constructed from the high and low exponential growth questions. It is however plausible that the order in which these questions are asked matters, for instance if people are able to learn (though there is no feedback). To abstract from these effects, we randomize the order of questions, where we treat the high and low exponential growth questions as a block. That is, participants are either first asked the high and low exponential growth questions (in random order) and then asked the mitigation question, or vice versa. An advantage of this design is that it allows for both within and across subject comparisons.

The experiment took place online on 25 and 26 March 2020. Subjects were university students in non-STEM fields, there were 111 subjects in Group D and 116 subjects in Group R. The experiment was conducted in German. All administration and subject contact was handled by the Decision Sciences Laboratory of ETH Zurich. Subjects were paid a non-contingent participation fee of CHF 10 (about EUR 9.50/USD 10 at the time of the study).

## Results

### Demographics and awareness of exponential growth bias

Before discussing our substantive findings, it might be useful to have an impression of who the subjects are. The demographic data was elicited at the end of the study, in particular after the exponential growth and mitigation questions. The median subject in the study is 23 years old, the youngest subject 19 years, with the 1st quartile at 22 years, the 3rd quartile at 25 years and the oldest subject 27 years of age (one subject declined to self-report age). 25% of subjects are male, 2% declined to state their gender or indicated a non-binary gender, and the remaining 73% are female. 70% are students at the University of Zurich, 21% at the Swiss Federal Institute of Technology (ETH Zurich), 8% at other institutions of higher education and 1% do not study. 70% are enrolled in a Bachelor’s program, 26% in a Master’s program, and 2% in a doctoral program (1% do not study). Fields of study that account for more than 10% of subjects are law (20%), medicine (19%), and architecture (12%). Students in STEM fields were excluded from the study. When asked to self-evaluate their mathematical abilities on a 5-item Likert scale, 4% of subjects reported their mathematical abilities as very bad, 19% as bad, 46% as average, 26% as good, and 4% as very good (1% declined to answer).

At the end of the study, prior to the demographic questions, subjects are asked whether they think others in the study are likely to underestimate, estimate approximately correctly, or overestimate exponential growth. With this, we aim to investigate whether our subjects are aware of the phenomenon of exponential growth bias (without needing to know the technical term “exponential growth bias”). To do this, the high exponential growth question in the respective frame is again displayed, and subjects are asked to indicate how they thought most other subjects answered, as Table 4 shows.

**Table 4:** Eliciting beliefs about the prevalence of exponential growth bias

*4a. Parameterization in terms of the daily growth rate (Group R)*
Earlier in the study, you answered the following question: “In a country, 974 people have been infected so far. The number of infected people grows by 26% per day. How many people will be infected in 30 days?” Which statement in your opinion best captures the answers of most other participants? □ The answers of most participants were far too low. □ The answers of most participants were too low. □ The answers of most participants were approximately correct. □ The answers of most participants were too high. □ The answers of most participants were far too high
*4b. Parameterization in terms of doubling time in days (Group D)*
Earlier in the study, you answered the following question: “In a country, 974 people have been infected so far. The number of infected people doubles every 3 days. How many people will be infected in 30 days?” Which statement in your opinion best captures the answers of most other participants? □ The answers of most participants were far too low. □ The answers of most participants were too low. □ The answers of most participants were approximately correct. □ The answers of most participants were too high. □ The answers of most participants were far too high

We find that in both groups most subjects are aware of exponential growth bias: 83% of subjects in Group R and 91% of subjects in Group D believe that most other subjects underestimate or strongly underestimate the number of cases after a month of exponential growth ($p=0.05$ for the difference in the shares).

When interpreting the following data, it might be helpful to have in mind a picture of a typical subject in the study: She would be a woman in her twenties enrolled in law, medicine or architecture at a leading Swiss university. She would rate her math skills as average, and believe that most of her peers in the study are subject to exponential growth bias.

### Parameterization and exponential growth bias

**Fig. 1:**
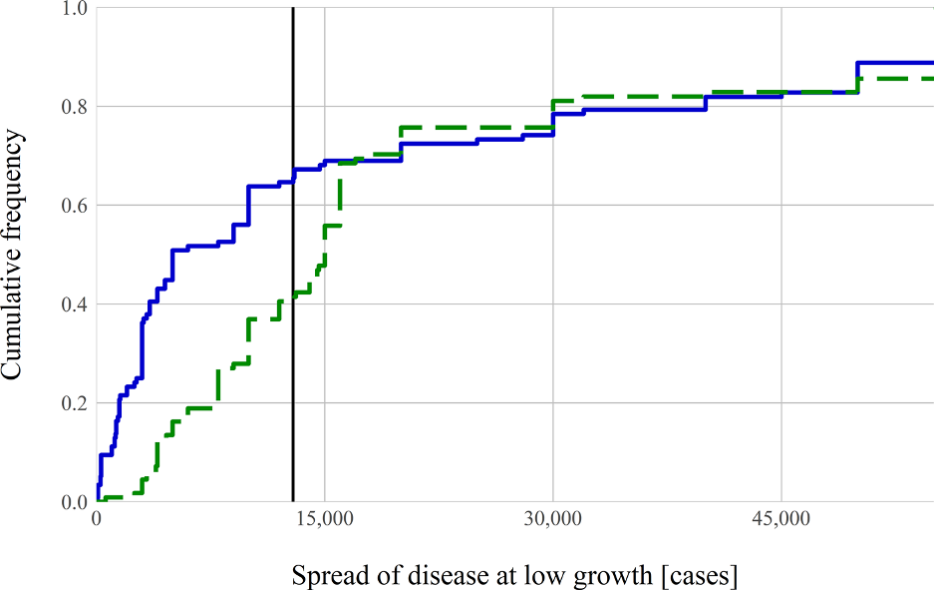
Effect of framing on exponential growth bias—low growth rate. Cumulative distribution functions of answers. The *blue solid line* shows answers from subjects who receive the information about growth in terms of the daily growth rate of 9% (Group R, *n* = 116). The *green dashed line* shows answers from subjects who receive the information about growth in terms of the doubling time of 8 days (Group D, *n* = 111). The *thick black vertical line* indicates the true value of about 13,000 cases

Let us now discuss our investigation of the prevalence of exponential growth bias in our subject pool in the context of infectious disease spread, and of how framing affects this bias. In the low exponential growth question, the true number of cases after 30 days in the country is around 13,000 cases. The median answer by subjects in Group R, who were communicated exponential growth by means of growth rates is 5000 cases. The median answer of subjects in Group D, where growth was communicated in terms of doubling times is 15,000 cases. Hence, the median answer in the frame using doubling times is closer to the correct amount ($$p< 10^{-5})$$)^3^. Turning to the fraction of subjects who underestimate exponential growth, i.e. who exhibit exponential growth bias, we find that 65% of subjects underestimate exponential growth when the growth rate is used (Group R), compared to only 41% of subjects who underestimate it when doubling time is used (Group D). This difference is statistically significant $$(p< 10^{-3})$$. Fig. 1 shows the distribution of answers to the low exponential growth question in both groups.

**Fig. 2:**
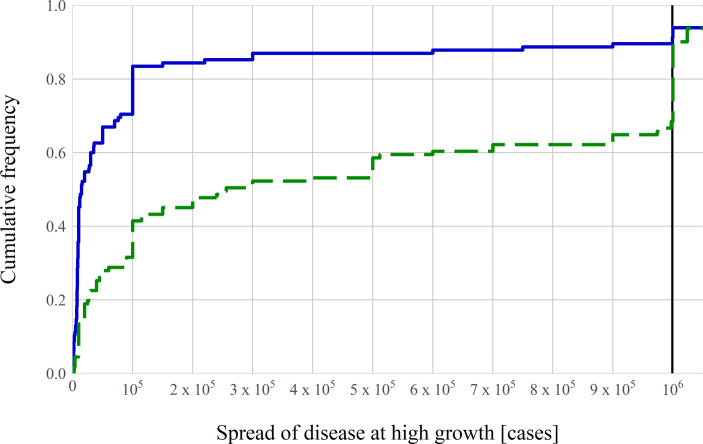
Effect of framing on exponential growth bias—high growth rate. Cumulative distribution functions of answers. The *blue solid line* shows answers from subjects who receive the information about growth in terms of the daily growth rate of 26% (Group R, *n* = 115). The *green dashed line* shows answers from subjects who receive the information about growth in terms of the doubling time of 3 days (Group D, *n* = 111). The *thick black vertical line *indicates the true value of about 1 million cases

Turning to the exponential growth questions with high growth, the correct answer is that after 30 days of exponential disease spread, there would be about one million cases in the country. Fig. 2 shows the distribution of answers to the high exponential growth question in both frames. The median answer by subjects in Group R, who receive the information on exponential growth in terms of the growth rate, is 15,000 cases. In Group D, who receive the information in terms of doubling time, the median answer is 256,000 cases. Hence, the median subject in both groups drastically underestimates the spread of the infectious disease, and the extent of exponential growth bias differs. In the growth rate framing, 90% of subjects exhibit exponential growth bias. 67% of subjects do so in the doubling time framing. In summary, we find that exponential growth bias is highly prevalent here, but framing the scenario using doubling times facilitates understanding: the share of subjects that exhibit the bias is lower (*p* < 10^−4^), and the median answer in that frame is closer to the correct amount (*p* < 10^−10^).

### Beliefs and bias when the growth rate changes

Phenomena of exponential growth are often embedded in complex problems. One might say that problems appear as *Textaufgaben*, word problems. Both the understanding of and formal translations of such problems typically demand more than simply calculating the value of an exponential function. For instance, such a more complex problem occurs when a decision-maker is given the option of an increase (or decrease) of the growth rate of an exponential function in exchange for some benefit or cost. An example of such a trade-off could be an offer by a bank offering lower fees in the exchange of slightly higher interest rates on a mortgage. Another example of a natural occurrence of such a trade-off is the adoption of non-pharmaceutical interventions during the Covid-19 pandemic: on the one hand, these interventions such as social distancing, the dialling down of social or economic life, or travel restrictions are individually and socially costly, but on the other hand they can be seen as decreasing the exponential growth of infections. In both of these examples, a decision-maker does not only need to understand an exponential growth process, but also the impact of a change in its growth rate.

Our experiment investigates how subjects perceive a change in exponential growth using the mitigation question. As the mitigation question is constructed from the high and low exponential growth questions, a natural hypothesis is that a subject’s answer to the mitigation question is about equal to the difference in her answers to the high and low exponential growth questions. Under this hypothesis, bias in the answer to the mitigation question should be fully accounted for by bias in the answers to the exponential growth questions. However, subjects’ intuition need not conceptualize the answer to the mitigation question in this way. Subjects are not instructed to take such an approach.

**Fig. 3:**
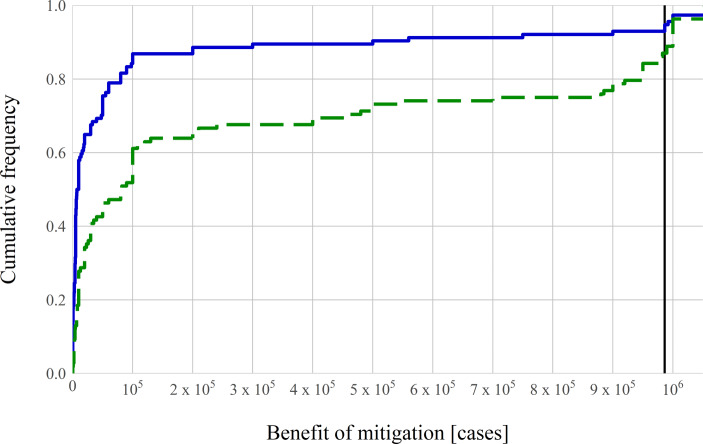
Effect of framing on mitigation bias. Cumulative distribution functions of answers. The *blue solid line* shows answers from subjects who receive the information about the difference in growth rates in terms of the daily growth rate of 26% resp. of 9% (Group R, *n* = 114). The *green dashed line* shows answers from subjects who receive the information about growth in terms of the doubling time of 3 days resp. of 8 days (Group D, *n* = 108). The *thick black vertical line *indicates the true value of about 986,000 cases

The correct answer to the mitigation question is that about 985,000 cases could be avoided with the mitigation measures. In both frames, most subjects drastically underestimate these potential benefits: the median answer in Group R, who were given the information in terms of the growth rate, is 8600 cases avoided. The median answer in Group D, who were given the information in terms of doubling times is 82,000 cases. Hence, the median answer in the group using doubling times exhibits less bias than the median answer in the group using growth rates (*p* < 10^−6^). 94% of subjects in Group R, and 87% of subjects in Group D underestimate the impact of the mitigation measures, that is they exhibit what we term *mitigation bias*: the underestimation of the effect of lowering the exponential growth rate. These shares are not statistically significantly different at the 99%-level. Fig. 3 shows the distribution of answers to the mitigation question in both frames.

### Comparing mitigation bias and exponential growth bias

Our subjects underestimate exponential growth in the context of infectious disease spread. Exponential growth bias is more prevalent when the disease spreads faster. Bias becomes even more prevalent when subjects are asked for the impact of a change in the exponential growth rate. Regardless of how the information is communicated, the fraction of biased subjects is highest for the mitigation question, second highest for the high exponential growth question and smallest for the low exponential growth question. Framing affects exponential growth bias: the fraction of biased subjects is always lower when exponential growth is communicated in doubling times rather than growth rates. Table 5 provides an overview of these results.

**Table 5:** Overview of intuitive beliefs

	Parameterization			
	Growth rate (Group R)		Doubling Time (Group D)	
	Share biased	Median	Share biased	Median
*Low exponential growth question* Group R: 9% daily growth, Group D: doubling time 8 days	65%	5000 [12,923]	41%	15,000 [13,105]
	*n* = 116		*n* = 111	
*High exponential growth question* Group R: 26% daily growth, Group D: doubling time 3 days	90%	15,000 [999,253]	67%	256,000 [997,376]
	*n* = 115		*n* = 111	
*Mitigation question* Group R: 26–9% daily growth Group D: doubling time 3–8 days	94%	8600 [986,330]	87%	82,000 [984,271]
	*n* = 114		*n* = 102	

Numbers in brackets give the true value

**Fig. 4:**
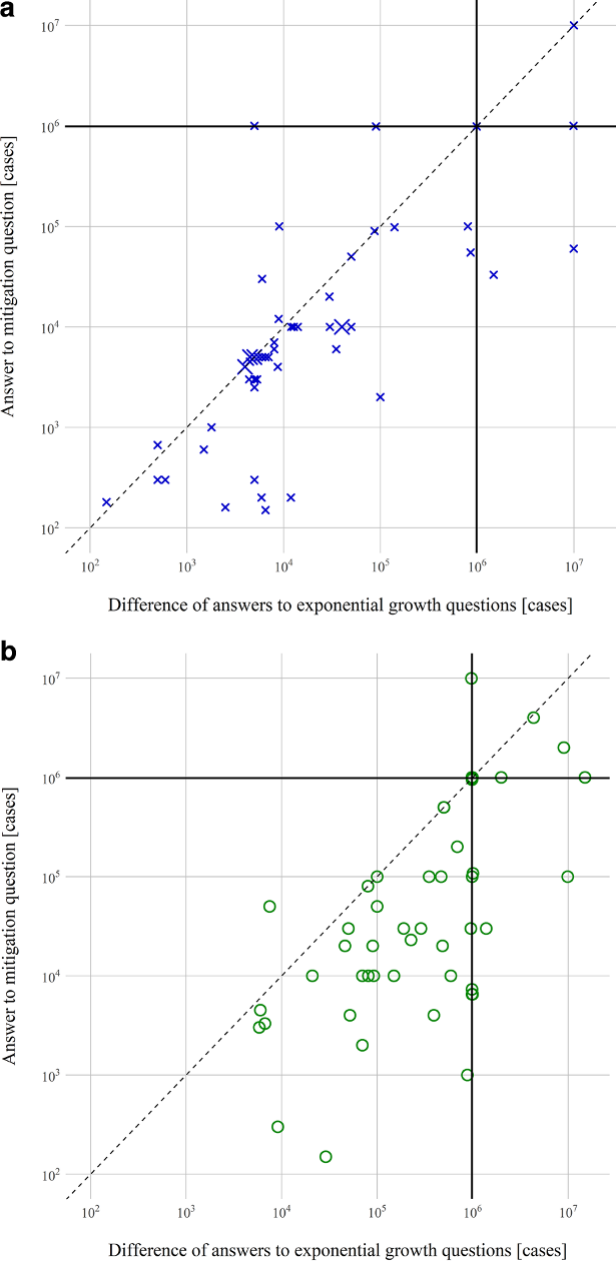
Relation of exponential growth bias and mitigation bias. Answers to the mitigation question plotted against the difference in answers to the exponential growth questions for Group R (**a**, *blue crosses*) and for Group D (**b**, *green circles*). *Solid lines* depict the correct answers: 986,330 cases avoided in Group R, 984,271 cases avoided in Group D. Answers on the *dashed line* can be fully explained by the answers to the exponential growth questions. *Larger symbols* correspond to multiple identical answers. *Axes* are capped. Data points with non-positive values are excluded. *n* = 54 in Group R, *n* = 51 in Group D

Mitigation bias is more prevalent than exponential growth bias. As the study design allows for within subject comparisons, we can investigate this further: compare each subject’s answer to the mitigation question to the difference in her answers to exponential growth questions. For 23% of subjects in Group R and 15% of subjects in Group D, the answer to the mitigation question is exactly equal to the difference in answers to the exponential questions. For these subjects, exponential growth bias can fully explain mitigation bias. Of the remaining subjects for whom exponential growth bias cannot fully explain mitigation bias, 75% of subjects in Group R and 66% of subjects in Group D give an answer to the mitigation question which is smaller than what would be implied by their answers to the component questions. For these subjects, mitigation bias is more severe than what exponential growth bias would suggest. The order in which a subject answers the mitigation vs. the exponential growth questions is randomized. In natural settings, people are typically not first primed with the component exponential questions, hence Fig. 4 restricts attention to subjects who see the mitigation question first. For these subjects, it plots their answers to the mitigation question against the difference in the answers to the exponential growth questions.

If the bias in the answer to the mitigation question is only due to exponential growth bias per se, then we have $m_{i}=h_{i}-l_{i}$, where *m*_*i*_ is individual i’s answer to the mitigation question, *h*_*i*_ her answer to the high exponential growth question and *l*_*i*_ to the low exponential growth question. To gauge to what extent this matches the data, specify the linear model $m_{i}=\delta (h_{i}-l_{i})$. Let *m, h*, and *l* be the true values and consider the null hypothesis that mitigation bias is no more severe than what exponential growth bias would predict, i.e. $m-m_{i}\leq \left(h-l\right)-(h_{i}-l_{i})$, or $\delta \geq 1$. This hypothesis is rejected at $p< 10^{-7}$ in the parameterization with growth rates, and at $p< 10^{-5}$ in the parameterization with doubling times, as Table 6 shows. These findings mean that exponential growth bias has bigger effects than one might think. After all, in many situations, the choice individuals face is not between exponential growth and no growth, but between different speeds of exponential growth.

**Table 6:** Mitigation bias and exponential growth bias

	Parameterization			
	Growth rates		Doubling times	
	Question(s) shown first		Question(s) shown first	
–	**Mitigation**	Exponential	**Mitigation**	Exponential
*Difference exp. questions* (Std. error)	0.37*** **(0.06)**	0.69*** (0.02)	0.14*** **(0.03)**	0.81*** (0.05)
Adj. R^2	**0.39**	0.95	**0.27**	0.87
*n*	**50**	48	**49**	35

The answer to the mitigation question is regressed on the difference between the answers to the high and the low exponential growth question

Only individuals are considered whose answers are positive, whose answer to the high exponential growth question is larger than the answer to the mitigation question and the low exponential growth question

One outlier $(> 10^{8}$) is excluded

Our preferred specification is in bold

***indicates *p* < 0.001

## Discussion and conclusion

Reducing exponential growth bias faces a two-step challenge. First, an individual must realize that a process is exponential. Second, she needs to realize that she is likely biased and that her bias is likely larger than she imagines, and then rationally over-ride her intuition. Recognition of exponential growth is hindered by three popular misconceptions: The first misconception sees growth as either linear or exponential, the second one views unboundedness as defining exponential growth, and the third one alleges that exponential growth is always very fast. The first misconception is often associated with pejorative use of the term linear as in *linear thinker* or *linear thinking*. Linear approximations are simple, and predict reasonably well in many problems. Exponential approximations do so in others, and yet other functional forms in other problems. Students should be taught that model selection is important, and be exposed to a variety of candidate functional forms, e.g., linear, exponential, logarithmic, logistic or polynomial growth. To counteract the second misconception of unboundedness defining exponential functions, it may be helpful to point out to students that (increasing) linear functions, quadratic or logarithmic functions all grow beyond any bound. The third misconception, the belief that exponential growth is per se very fast, is potentially dangerous. People operating under this misconception may fail to identify exponential growth in its early stages. For instance, in the case of the Covid-19 pandemic, the number of new cases per day is initially very small and increases slowly—only to then increase speed. This example could be used to demonstrate to students that slow growth may still be generated by an exponential process. Relatedly, students should appreciate that exponential functions are strongly convex.

Once an individual has recognized that a process is exponential, her situation might be compared to someone who is confronted with an optical illusion and realizes it. In this paper, we find that exponential growth bias is prevalent and substantial even though most subjects are aware of exponential growth bias. Bias becomes even worse when a problem embeds exponential questions, as is the case in most real-world applications. Taken together with the fact that our subject pool is highly educated, this suggests that teaching about exponential growth and exponential growth bias is insufficient.

This paper provides evidence that communication of information in doubling times rather than growth rates decreases bias. This lends itself to recommendations for individuals, educators and policymakers. People should be wary of exponential growth bias, especially when receiving information in terms of growth rates. They might want to apply the rule of 72 to convert growth rates into doubling times (divide 72 by the growth rate in percent to obtain the approximate doubling time). Educators could teach this heuristic. Policymakers should realize that mandates to clearly disclose exponential growth, such as annualized percentage rates (APR), are likely not enough. They should consider mandates to communicate doubling times, and even final values due.

The data presented in this article reminds us of the simple fact that human intuition is limited. Individuals therefore need to override their intuition and employ mathematics. We hope that our findings encourage more research into approaches like converting growth rates into doubling times that may assist intuition and lessen the problem of exponential growth bias.

## Footnotes

1 Most or all of these phenomena occur against the backdrop of a limited carrying capacity. Hence, if the focus is on the long run, logistic models may be more appropriate. For an educational exposition of the use of exponential vs. logistic models illustrated in the context of population ecology see Vandermeer [22].

2 In the experiment, there are two additional randomized groups of subjects. These subjects receive logarithmic questions. The results are reported in Schonger and Sele [19].

3 To test whether a difference in medians is statistically significant, we use the asymptotic two-sample Brown-Mood median test.

## References

[1] Almenberg J, Gerdes C (2012). Exponential growth bias and financial literacy. Appl. Econ. Lett..

[2] BBC Future: Exponential growth bias: the numerical error behind Covid-19 (2020). https://www.bbc.com/future/article/20200812-exponential-growth-bias-the-numerical-error-behind-covid-19, Accessed 20 Aug 2020

[3] Bierman H (1989). The Allais paradox: a framing perspective. Behav. Sci..

[4] Christandl F, Fetchenhauer D (2009). How laypeople and experts misperceive the effect of economic growth. J. Econ. Psychol..

[5] Dalton, C.: I’m an ER doctor. Please take coronavirus seriously (2020). https://www.theguardian.com/commentisfree/2020/mar/20/er-doctor-coronavirus-exponential-growth, Accessed 20 Mar 2020

[6] Dante, Kline A (2000). Paradiso, Canto XXVIII. Poetry in Translation, Early 14th century.

[7] Foltice B, Langer T (2018). Exponential growth bias matters: evidence and implications for financial decision making of college students in the USA. J. Behav. Exp. Finance.

[8] Goda GS, Manchester CF, Sojourner AJ (2014). What will my account really be worth? Experimental evidence on how retirement income projections affect saving. J. Public Econ..

[9] Kahneman D, Tversky A (1979). Prospect theory: an analysis of decision under risk. Econometrica.

[10] Kashima Y, Maher P (1995). Framing of decisions under ambiguity. J Behav. Decis. Mak..

[11] Keller LR (1985). The effects of problem representation on the sure-thing and substitution principles. Manag. Sci..

[12] Königsheim C, Lukas M, Nöth M (2018). Individual preferences and the exponential growth bias. J. Econ. Behav. Organ..

[13] Khallikan I (1868). Biographical Dictionary.

[14] Lammers J, Crusius J, Gast A (2020). Correcting misperceptions of exponential coronavirus growth increases support for social distancing. Proc. Natl. Acad. Sci. U.S.A..

[15] Levy MR, Tasoff J (2016). Exponential growth bias and lifecycle consumption. J. Eur. Econ. Assoc..

[16] Levy MR, Tasoff J (2017). Exponential-growth bias and overconfidence. J. Econ. Psychol..

[17] Macdonell AA (1898). Art. xiii.—The origin and early history of chess. J. Royal Asiatic Soc. G. B. Irel..

[18] Mckenzie CRM, Liersch MJ (2011). Misunderstanding savings growth: implications for retirement savings behavior. J. Mark. Res..

[19] Schonger M, Sele D (2020). How to better communicate the exponential growth of infectious diseases. PLoS ONE.

[20] Stango V, Zinman J (2009). Exponential growth bias and household finance. J. Finance.

[21] Tversky A, Kahneman D (1981). The framing of decisions and the psychology of choice. Science.

[22] Vandermeer J (2010). How populations grow: the exponential and logistic equations. Nat. Educ. Knowl..

[23] Wagenaar WA, Sagaria SD (1975). Misperception of exponential growth. Atten. Percept. Psychophys..

[24] Wagenaar WA, Timmers H, Burkhardt DF, Ittelson WH (1978). Intuitive prediction of growth. Environmental Assessment of Socioeconomic Systems.

[25] Wagenaar WA, Timmers H (1979). The pond-and-duckweed problem: three experiments on the misperception of exponential growth. Acta Psychol..

[26] Zeit: Schach und das Virus: Warum das Ausbreitungstempo bei Corona so entscheidend ist (2020). https://www.zeit.de/news/2020-03/12/warum-das-ausbreitungstempo-bei-corona-so-entscheidend-ist, Accessed 12 Mar 2020

